# Recent Advances in Efficient Lutein-Loaded Zein-Based Solid Nano-Delivery Systems: Establishment, Structural Characterization, and Functional Properties

**DOI:** 10.3390/foods13142304

**Published:** 2024-07-22

**Authors:** He Han, Ying Chang, Yan Jiao

**Affiliations:** College of Food and Bioengineering, Qiqihar University, Qiqihar 161006, China; 03500@qqhru.edu.cn (H.H.); 02512@qqhru.edu.cn (Y.C.)

**Keywords:** lutein, zein, nano-delivery system, structural characterization, functional properties

## Abstract

Plant proteins have gained significant attention over animal proteins due to their low carbon footprint, balanced nutrition, and high sustainability. These attributes make plant protein nanocarriers promising for applications in drug delivery, nutraceuticals, functional foods, and other areas. Zein, a major by-product of corn starch processing, is inexpensive and widely available. Its unique self-assembly characteristics have led to its extensive use in various food and drug systems. Zein’s functional tunability allows for excellent performance in loading and transporting bioactive substances. Lutein offers numerous bioactive functions, such as antioxidant and vision protection, but suffers from poor chemical stability and low bioavailability. Nano-embedding technology can construct various zein-loaded lutein nanodelivery systems to address these issues. This review provides an overview of recent advances in the construction of zein-loaded lutein nanosystems. It discusses the fundamental properties of these systems; systematically introduces preparation techniques, structural characterization, and functional properties; and analyzes and predicts the target-controlled release and bioaccessibility of zein-loaded lutein nanosystems. The interactions and synergistic effects between Zein and lutein in the nanocomplexes are examined to elucidate the formation mechanism and conformational relationship of zein–lutein nanoparticles. The physical and chemical properties of Zein are closely related to the molecular structure. Zein and its modified products can encapsulate and protect lutein through various methods, creating more stable and efficient zein-loaded lutein nanosystems. Additionally, embedding lutein in Zein and its derivatives enhances lutein’s digestive stability, solubility, antioxidant properties, and overall bioavailability.

## 1. Introduction

**Figure 1:**
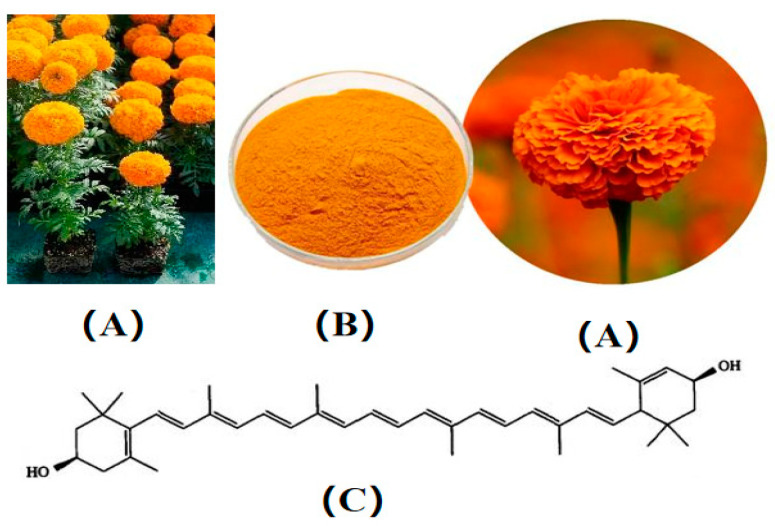
*Tagetes erecta* (**A**), lutein commodities (**B**), chemical structure of lutein (**C**).

The lutein (C_40_H_56_O_2_) is a carotenoid with a 40-carbon skeleton structure composed of two 20-carbon precursors joined head-to-head. Its molecular structure, shown in Figure 1, reveals a diverse arrangement of conjugated double bonds with both linear and cyclic configurations [1]. Lutein, primarily extracted from marigolds, is a fat-soluble pigment belonging to the group of oxygenated carotenoids, also known as photosensitive and macular pigment [2]. Widely recognized by consumers for its unique golden color, lutein serves as a popular colorant. Numerous studies have demonstrated lutein’s strong antioxidant and anti-inflammatory properties [3,4,5,6]. Experiments conducted in vivo and in vitro have also revealed its neuroprotective, retinal protective, and lipid-lowering functions, with its eye protection capabilities garnering significant attention [7,8,9,10,11,12,13,14]. Recent research indicated that lutein is one of the most important carotenoids in human serum, providing a strong antioxidant effect and exhibiting inhibitory effects on cancer and tumors [15]. Dietary intake of lutein is considered safe, with no side effects observed from long-term supplementation of 30–40 mg/d. This excellent bioactivity and safety profile have led many companies to use lutein as a preferred raw material for “clean label” products [16]. However, the use of lutein is limited by its poor solubility, stability, and bioavailability [17]. It is particularly unstable during food storage due to oxidation, which reduces its biological activity and causes undesirable fading.

Lutein’s strong antioxidant properties contribute to its decomposition when exposed to light, heat, and oxygen, making preservation difficult. Additionally, lutein’s structure complicates its water solubility and bioavailability. The high hydrophobicity of lutein’s C-40 isoprene structure reduces its solubility and permeability across intestinal cells, limiting intestinal absorption to 10–15% [18]. Since the body cannot synthesize lutein, its absorption relies on dietary intake from fruits, vegetables, and synthetic supplements [19]. The complexation of lutein with food substrates further limits its bioavailability.

To address these challenges, food and nutrition scientists are developing effective strategies. The concept of “new nano-delivery systems” has gained traction in the food industry and is widely applied in special medical, health, and functional foods [20]. Nanocarriers have been found to improve lutein’s bioavailability. Nanocapsules, a primary delivery system, enhance the bioavailability and efficacy of hydrophobic drugs and nutrients, such as curcumin [21] and taxol [22]. Furthermore, nanoparticles can deliver antioxidants directly to tumor cells more effectively [23]. Therefore, the nano-embedded delivery of lutein is essential for improving its bioavailability and producing lutein-functional foods, marking a future development trend for lutein products.

## 2. Structural Properties and Transport and Transport Functions of Zein

### 2.1. Physicochemical Properties of Zein

Zein is the major storage protein of maize and one of the few edible hydrophobic polymers. Based on solubility and amino acid sequence homology, Zein can be classified into α-, β-, γ- and δ-zearalbumin, with α-zearalbumin being the most abundant, accounting for more than 80% of the total Zein [24,25], as shown in Table 1.

Zein is insoluble in water but soluble in ethanol, high concentrations of urea, alkaline pH (>11), or anionic surfactants [26]. The solubility of Zein is attributed to its composition of over 50% hydrophobic amino acids [27]. Glutamine-rich corners link the protein helix segments together in an antiparallel fashion [28]. The top and bottom loops of Zein, containing glutamine, are hydrophilic, while the alpha-helix-containing surface is hydrophobic. This amphiphilic nature enables Zein to self-assemble.

### 2.2. Embedding and Transport Functions of Zein

Zein has proven to be an excellent material for the manufacture of nanoparticle delivery systems such as films [29], coatings [30], and emulsions [31], significantly improving the stability, bioactivity, and bioavailability of core materials [32,33,34].

The polarity of the solution can alter the solubility of Zein, and its amphiphilic properties drive a spontaneous aggregation phenomenon known as the self-assembly process. This process can transform Zein from a random to an ordered conformation. Zein’s excellent self-assembly ability has been utilized to prepare bioactive substance carriers, laying a solid foundation for the food and pharmaceutical industries [35]. Shi et al. encapsulated resveratrol in Zein nanoparticles, demonstrating that the encapsulated resveratrol exhibited superior antioxidant and anticancer properties in vitro and higher bioaccessibility than free resveratrol [36]. Wang et al. investigated the colloidal complexation of corn alcohol-soluble protein hydrolysate (ZH) with tannic acid (TA) and utilized the ZHTA complex to transport algal oil. Experiments showed that the ZHTA complex had significant physical stability and algal oil encapsulation efficiency and imparted high antioxidant properties to the ZH-TA complex [37]. Spinella et al. prepared colloidal composite nanoparticles using Zein, sodium caseinate, and pectin for eugenol encapsulation. The results indicated that the nanocomposite particles had good redispersibility, excellent stability after 56 days of storage, and no bacterial growth at room temperature, demonstrating superb microbial stability [38]. Ahmadzadeh et al. used Zein loaded with lutein as the inner flow material and corn starch slurry as the outer flow material in coaxial extrusion 3D printing technology. They found that when lutein was encapsulated in the starch/zein gel at 25 °C for 21 days, only 39% of the lutein degraded, compared to a 78% degradation rate of the lutein monomer. Similar improvements were observed after 21 days of storage at 50 °C [39]. These findings illustrate that nutrients, bioactive substances, and drugs, especially hydrophobic substances, can be encapsulated in zein-based delivery systems to improve their stability and bioactivity.

## 3. Establishment of Lutein Nanoparticle Delivery System Supported by Zein Carrier

### 3.1. Preparation of Zein-Loaded Lutein Nanoparticles

The self-assembly characteristics of Zein enable several technical methods for preparing zein–lutein nanocarriers, including nano-lipidation preparation technology, antisolvent nanoparticle preparation technology, and supercritical fluid-enhanced solution dispersion technology.

#### 3.1.1. Nano-Lipidation Technology

**Figure 2:**
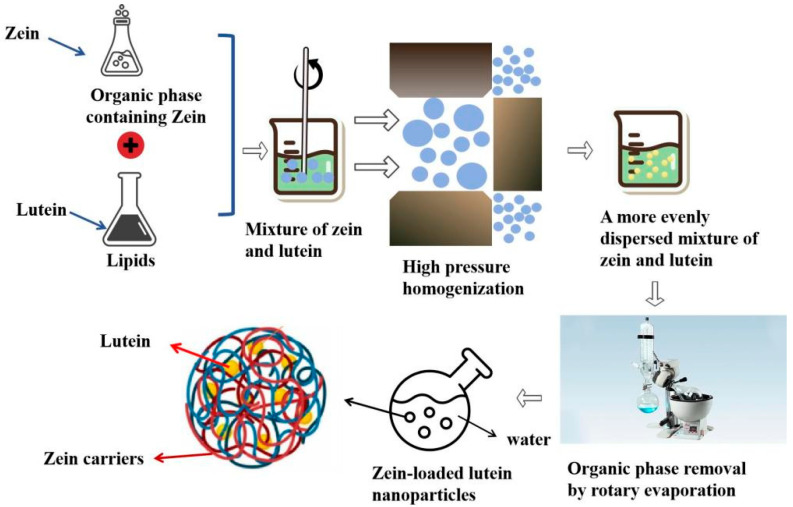
Preparation of lutein-loaded Zein nanoparticles by nano-lipidation.

Lipid nanocarriers can improve drug stability, achieve targeted drug delivery, and enhance bioavailability [40]. The preparation process is illustrated in Figure 2. In liposomes, the lipid phase comprises solid and liquid lipids, forming a less ordered crystalline or amorphous solid structure. The reduced water content in liposomes minimizes the rapid release of loaded compounds during storage. Additionally, they improve the bioavailability of lipophilic bioactive compounds due to their excellent physical stability. These properties make liposomes a suitable carrier for bioactive ingredients, garnering significant interest from researchers across various fields [41]. Liu et al. developed three types of lipid carriers loaded with lutein: nanoemulsions, solid lipid nanoparticles, and nanostructured lipid carriers [42].

#### 3.1.2. Anti-Solvent Nanoparticles Technology

**Figure 3:**
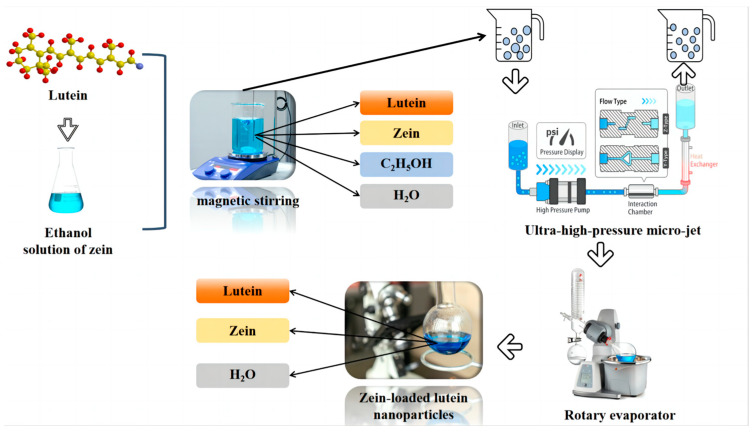
Preparation of maize alcohol soluble protein-loaded lutein nanoparticles by anti-solvent method.

The anti-solvent method involves dissolving Zein in a 60–95% ethanol solution and heating the Zein solution to evaporate the ethanol [43]. During this process, the polarity of zein changes from hydrophobic to hydrophilic, and the protein molecules assemble into nanoparticles through hydrophobic interactions. The α-helix in Zein molecules transforms into β-folding, encapsulating the central active ingredient [44]. As shown in Figure 3, after the purification of Zein, zein–lutein nanoparticles can be successfully synthesized using the anti-solvent precipitation method, with ascorbic acid added as an antioxidant in the aqueous dispersion. Researchers have prepared zein nanoparticles to encapsulate lutein using solvent-induced nanoprecipitation, finding that the nanoparticles are significantly digested into peptides under intestinal digestion conditions, increasing lutein’s digestive stability. The addition of Zein increased lutein’s digestive stability by 58% compared to water-based lutein dispersants, suggesting that Zein provides physical protection for encapsulated lutein under gastric conditions [45]. Additionally, it was found that the maximum encapsulation rate of Zein for lutein was 81.0%, indicating that Zein can embed lutein through hydrogen bonding and hydrophobic interactions, thereby protecting lutein. The secondary structure of Zein did not change significantly, and the stability and dispersibility of lutein were improved, enhancing the bioactivity and utilization of lutein [46].

#### 3.1.3. Supercritical Carbon Dioxide Preparation Technology

**Figure 4:**
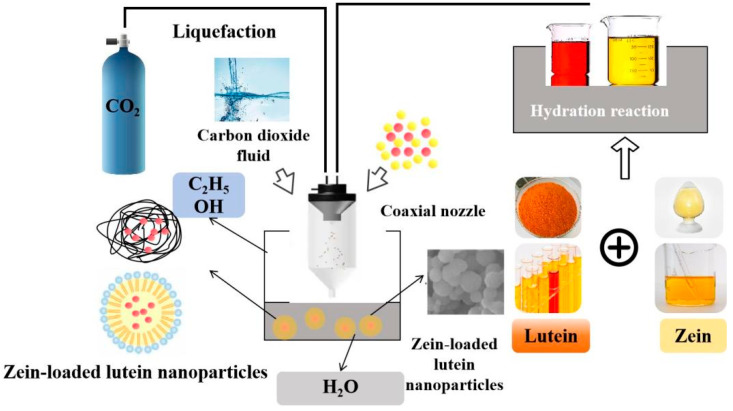
Preparation of zein-loaded lutein nanoparticles by supercritical fluid method.

In supercritical fluids, nanoparticles are produced by mechanically controlling process parameters such as pressure, temperature, ratio, and solution flow rate [47]. The supercritical fluid-enhanced solution dispersion technology utilizes high-speed supercritical fluid through a special coaxial channel double-layer nozzle to disperse the introduced lutein and Zein solution into small droplets. These droplets are then sprayed into a sedimentation tank, reducing their particle size, accelerating dispersion and expansion rates, and ensuring synchronous, rapid mixing of the atomized droplets. Factors such as the Reynolds number, solution flow distance, SC-CO_2_, and nozzle structure influence particle size and morphology. The high-velocity SC-CO_2_ passing through the nozzle provides kinetic energy to the solution exiting the nozzle, causing it to disperse and break into very small droplets, increasing the mixing of the droplet with the SC-CO_2_. Simultaneously, SC-CO_2_ diffuses into the droplet as an antisolvent, supersaturating the solution and causing finer particles to precipitate. This process leverages the chemical and mechanical properties of the supercritical fluid to enhance the spraying effect, ultimately achieving micro-pulverization [48]. The process principle is illustrated in Figure 4. Studies have shown that lutein/zein nanoparticles can be successfully prepared using supercritical antisolvent technology. Optimal conditions for preparing smaller and more regular spheres include lower temperatures, slower solution flow rates, and higher pressures [49]. Under conditions of 10 MPa pressure, a lutein/zein ratio of 1:18 (*w*/*w*), a solution flow rate of 1.0 mL/min, and a temperature of 45 °C, lutein/Zein nanoparticles with a narrow particle size range and controlled release ability were obtained. The release curve of lutein indicates a release profile close to zero-order, demonstrating the significant role of the nanoparticles in the controlled release of lutein.

### 3.2. Using Modified Zein to Obtain Lutein-Supported Nanoparticles

Modification of zein was carried out. One method was to improve the molecular flexibility of zein by acid-induced deamidation and to prepare flexible zein-loaded lutein nanoparticles [50]. The results showed that the distribution of flexible Zein nanoparticles was more orderly and formed a network structure, and the content of surface or total lutein of flexible zein nanoparticles was higher than that of natural zein-loaded lutein. This is because the α-helix content of flexible zein molecules decreases and the content of irregular curls increases, and the disorder of protein molecules is conducive to the adsorption of proteins and lutein, while the change in the secondary structure of flexible zein is attributed to the change of amino acid composition due to acid-induced deamidation reaction. The second is biological enzymatic modification [51], which uses various enzymes to modify the structure of zein, improving one or more of its physical and chemical properties to achieve the effect of enhancing certain functional properties, making it more suitable as an effective carrier of lutein. Some amphiphilic proteolytic hydrolysates also have self-organising properties and are ideal natural emulsifiers. They have a higher solubility than the original proteins and help to improve the digestion and absorption of key active ingredients by the human body, thus improving bioavailability [52]. Studies have shown that the encapsulation rate of lutein by alkaline protease hydrolysis of zein is more than 85% due to the better binding ability of peptide and lutein. The solubility of lutein nanoparticles encapsulated in zein-derived peptide nanoparticles was significantly higher than that of zein–lutein and free lutein nanoparticles. The stability of lutein in zein-derived peptide nanoparticles was also improved in simulated gastric fluid and simulated intestinal fluid [53]. Zein was hydrolysed by a neutral protease to prepare zein–lutein nanoparticles. At the same time, the encapsulation rate of lutein nanoparticles after pepsin hydrolysis was 93.82%, and the hydrolysed Zein loaded with lutein had a smaller nanoscale size and dispersibility. In addition, the digestive stability and release of lutein from zein hydrolysate nanoparticles in simulated gastric fluid (SGF) and simulated intestinal fluid (SIF) were significantly improved [54].

### 3.3. Using Composite Zein to Obtain Lutein-Supported Nanoparticles

Although Zein, a natural nanocarrier, has irreplaceable advantages in food delivery systems and has been widely studied and used in various food nanocarriers, it has some limitations. The fragile secondary structure of zein results in poor thermal stability, sensitivity to acid and base changes, and susceptibility to degradation by human digestive enzymes. To overcome these challenges, researchers have designed composite nanoparticles and modified the functional properties of a single carrier by mixing different carrier materials to achieve better encapsulation effects. One approach involves adding polysaccharides to Zein to modify it, with different sugars producing varying effects. For example, researchers have used antisolvent precipitation to prepare zein/soluble soy polysaccharide complex nanoparticles to encapsulate lutein [55]. The encapsulation efficiency reached 80%, and the water solubility and chemical stability of lutein were greatly improved after encapsulation. The composite nanoparticles also exhibited excellent acid–base stability and salt stability. The bioavailability of encapsulated lutein (32.11%) was significantly higher than that of unencapsulated lutein (16.21%). Another study by Ma et al. involved preparing zein/tea saponin nanoparticles as carriers for lutein, which improved the acid–base stability, ionic stability, and thermal stability of lutein. Additionally, the redispersibility remained good after 15 days of storage [56]. Furthermore, Zein glycosylated by transglutaminase was used to prepare lutein-loaded Zein nanoparticles, significantly enhancing the release of lutein in vitro in the simulated gastrointestinal tract. Glycosylated Zein encapsulation also improved the antioxidant activity of lutein in vitro [57].

## 4. Structural Characterization of Zein-Loaded Lutein Nanoparticles

The properties of zein–lutein nanoparticles mainly include encapsulation efficiency, particle size, polydispersity index (PDI), zeta potential, and microscopic morphology. These characteristics influence the stability, rheological properties, and sensory appearance of colloidal dispersions. Physical and chemical properties can be characterized by techniques such as spectroscopy, light scattering, and microscopy. Additionally, the overall composition and structural properties can be further analyzed using infrared spectroscopy, X-ray diffraction, and nuclear magnetic resonance as shown in Table 2. The structural stability of the nanoparticles can be improved and enhanced through Zein structural modification and various modification technologies.

### 4.1. Particle Size, Potential Analysis and Detection of Zein-Loaded Lutein Nanoparticles

Dynamic light scattering (DLS) is the most common and widely used method for measuring particle size for zein-loaded lutein nanoparticles. DLS is a technique that determines particle size and size distribution by illuminating a particle sample with a beam of light and measuring the Doppler shift of the scattered light. The technique is based on the Brownian motion of particles in a liquid. By analyzing the correlation function of the intensity of the scattered light over time, the velocity of the particles is deduced, and the size of the particles is then calculated [61]. The zeta potential measures the effective charge on the surface of a nanoparticle. A higher zeta potential indicates more net positive or negative charges on the surface of the particles, leading to stronger electrostatic repulsion between particles and increased stability of the dispersion [62]. Additionally, the zeta potential helps determine if sufficient modifiers are encapsulating the nanoparticle surfaces, which helps identify the optimum mass ratio of Zein to modifier [63]. The particle size distribution is generally represented by the PDI. A PDI value of less than 0.3 for the prepared nanoparticle size indicates that the nanoparticle system has formed a good dispersion system.

### 4.2. Microstructural Analysis of Zein-Loaded Lutein Nanoparticles

Transmission electron microscopy (TEM), scanning electron microscopy (SEM), and atomic force microscopy (AFM) are three commonly used techniques to obtain information about nanoparticles.

SEM allows us to visualize the surface of any sample and obtain a three-dimensional image. AFM can determine the surface of a material on an atomic scale. Both SEM and AFM can provide information about the surface of a sample and display a three-dimensional image. However, AFM is less effective at examining the morphology of polysaccharides in isolation and is better suited for resolving subtle changes on highly smooth surfaces [64]. It should be noted that AFM imaging is performed on samples that have been dehydrated and dried, so the sizes reflected in the images may differ from DLS measurements taken in colloidal dispersions. TEM can provide information about the internal structure of a sample by producing a two-dimensional image but is limited to thin samples to allow electrons to pass through. Due to the low electron density of polysaccharides, TEM sometimes requires staining to clearly characterize their morphology.

Previous nanoparticle morphology studies showed that SEM is the most suitable tool for observing Zein/lutein nanoparticles with droplet sizes ranging from 100 to 1000 nm. Eaton et al. demonstrated that SEM is appropriate for large nanoparticles (diameter greater than 50 nm), while AFM and TEM provide accurate results for smaller nanoparticles [65].

A single characterization technique cannot clearly observe the microscopic morphology of nanoparticles, likely because nanoparticles often lack regular geometric shapes. Consequently, researchers prefer to combine several techniques for observation. The combination of DLS measurements and TEM observations confirmed that tea saponins coat the surface of Zein rather than Zein being encapsulated in the polymer network [56]. In the binary composite system of Zein and tea saponin, Zein nanoparticles exhibited a spherical structure but were uneven and slightly aggregated. In contrast, Zein/tea saponin nanoparticles (ZTSNPs) and Zein/tea saponin-supported lutein nanoparticles (ZTSLNPs) were smoother and better dispersed. Additionally, ZTSLNPs had a smaller spherical structure compared to ZTSNPs.

### 4.3. Chemical Structure Analysis of Zein-Loaded Nanoparticles by FTIR and XRD

Fourier Transform Infrared Spectroscopy (FTIR) and X-ray diffraction (XRD) are widely used to study the overall composition and structural properties of biomaterials. FTIR is particularly useful for investigating interactions in nanoparticles and efficiently identifying functional groups. FTIR employs a continuous wavelength light source to irradiate the sample. The sample molecules absorb light at specific wavelengths, and a Fourier transform is applied to obtain the sample’s single-beam spectrum. This process produces absorption peaks in the infrared spectrum at the wavelengths absorbed by the sample. The more light is absorbed, the lower the transmittance and the stronger the absorption peak intensity, which is expressed as absorbance or transmittance. Protein vibration produces characteristic infrared absorption bands, such as amide A, amide B, and amides I to VII [66]. Among these, amide I and II bands are crucial due to their sensitivity to the protein secondary structure. The peak position of amide I occurs in the region 1700 to 1600 cm^−1^ (predominantly C=O stretching), and the amide II band occurs in the region 1600 to 1500 cm^−1^ (C-N stretching coupled with N-H bending modes). The bands between 3100 and 3500 cm^−1^ correspond primarily to O-H stretching vibrations, particularly in proteins, where this absorption is generally attributed to the O-H vibrations of water molecules. However, this region may also be influenced by the stretching vibrations of free amino groups (-NH_2_) or amide I (-NH-) bonds in proteins [67,68]. The CH_2_ antisymmetric and symmetric stretching vibrations range from 3000 to 2800 cm^−1^ [69]. A shift in the band peak from 3100 to 3500 cm^−1^ usually indicates the appearance of hydrogen bonding. Changes in amide I and II bands usually signal shifts in the secondary structure of proteins and the formation of hydrogen bonds. The bands at 3000 to 2800 cm^−1^ provide information about hydrophobicity. Li et al. indicated that soybean polysaccharide (SSPS) exhibited a characteristic peak at 1042 cm^−1^ due to rhamnogalacturonic acid. This peak also appeared in zein/SSPS composite nanoparticles (ZSNPs), indicating the formation of composite nanoparticles between Zein and SSPS. As for lutein, the absorption band near 1532 cm^−1^ is attributed to the stretching vibration of C=C in the six-atom ring. After encapsulation, this typical lutein peak also appeared in the nanoparticles, proving that zein/SSPS composite nanoparticles could support lutein molecules [55].

XRD involves the incidence of a monochromatic X-ray on a crystal. The crystal consists of atoms arranged regularly within a cell, with distances between these atoms being of the same order of magnitude as the wavelength of the incident X-ray. Consequently, the X-rays scattered by different atoms interfere, resulting in strong X-ray diffraction in certain directions. The diffraction phenomenon is closely related to composition, crystal type, molecular bonding, and molecular configuration. XRD has been extensively used to study the state of encapsulated compounds, such as resveratrol [70], quercetin [67], curcumin [71], and CoQ10 [72], all of which were found to be in an amorphous form after encapsulation into nanoparticles [73]. In the study, multiple diffraction peaks of lutein between 5° and 30° indicate the presence of unencapsulated lutein in crystalline form. Zein nanoparticles showed two small peaks at 9.1° and 19.3°, respectively, while SSPS showed only one peak at about 18.6°. All diffraction peaks for these biopolymers were relatively flat, indicating that Zein and SSPS exist in amorphous structures. After encapsulation, the typical diffraction peak of lutein did not appear in the spectrum of zein/SSPS composite nanoparticles loaded with lutein, suggesting that lutein may be distributed in the nanoparticles in an amorphous state [55].

## 5. Functional Properties of Zein-Loaded Lutein Nanosystems

### 5.1. Stability of Zein-Loaded Lutein Nanoparticles

The use of lutein in the food industry is limited due to its instability and chemical changes during food processing. High temperatures, oxygen, light, and extreme pH can affect lutein’s integrity, necessitating its protection through encapsulation. Experiments have shown that lutein–zein nanoparticles were successfully synthesized using the anti-solvent method, with ascorbic acid added as an antioxidant. The results demonstrated that the photodegradation rate of lutein was significantly reduced. Compared to pure lutein dispersion, the photostability of lutein in the ascorbate-stabilized dispersion improved significantly, with relative stability increasing by about 25% [58]. A liquid–liquid dispersion method was used to synthesize lutein-containing zein nanoparticles stabilized by lecithin and Pluronic F127 surfactants [59]. After 10 h of UV irradiation, only 1.42% of the pure lutein dispersion remained, while at least 46.53% of the lutein protected by composite nanoparticles was retained. Nanoparticles prepared with tea saponin and Zein combined with lutein showed excellent stability in terms of ionic strength, storage, and thermal conditions. Previous studies indicated that zein/chondroitin sulfate nanoparticles became unstable at salt concentrations above 15 mmol/L, whereas ZTSLNPs remained stable at salt concentrations up to 400 mmol/L. After 12 days of storage, the retention rate of free lutein was less than 10%. However, over 15 days of storage, the lutein retention rate in ZTSLNPs was higher than 90%, further demonstrating the effectiveness of the delivery system in protecting lutein [56].

### 5.2. Solubility of Zein-Loaded Lutein Nanoparticles

The poor water solubility of lutein often limits its application in functional foods and beverages. Jiao et al. used solvent diffusion to prepare zein-derived peptide nanoparticles, significantly enhancing solubility and dispersibility to 0.655 mg/mL, a 12.84-fold increase compared to a free lutein suspension in distilled water [53]. This improvement may be attributed to the encapsulation of proteins, which inhibits lutein crystallization, decreases molecular fluidity, and increases solubility. The solubility and dispersibility of lutein also increased significantly with glucosamine. When glucosamine-glycosylated zein nanoparticles were produced by transglutaminase, the highest solubility reached 9.33 μg/mL. Zein binds to glucosamine through an enzymatic reaction, lowering the zeta potential, preventing nanoparticle aggregation, and improving hydrophobicity. As a result, the solubility and polydispersity of encapsulated lutein nanoparticles improved significantly compared to unmodified lutein-loaded zein nanoparticles and free lutein, with solubility increased more than 10-fold [57]. Li et al. found that the solubility of lutein in zein/soy polysaccharide nanoparticles increased by more than 30 times [55]. However, the poor redispersibility of lyophilized lutein-loaded nanoparticles was observed experimentally, possibly due to the formation of a thick layer of neutral sugar side chains from the soy polysaccharide on the surface of the protein particles. Different interaction mechanisms may exist between Zein and other polysaccharides, such as pectin or soy polysaccharides.

### 5.3. Antioxidant Activity of Zein-Loaded Lutein Nanoparticles

Oxidative stress is a significant cause of human aging and various diseases, which has led to increased interest in the screening, preparation, and application of antioxidants. Antioxidants are used in food processing and health foods, cosmetics, and drug development due to their functional roles. With some chemically synthesized antioxidants exhibiting toxic side effects, natural antioxidants have garnered attention for their safety. Chang et al. found that encapsulation of Zein and glycosylated Zein enhanced the antioxidant activity of lutein in vitro. These findings align with previous studies, suggesting that glycosylation of Zein can indeed protect and enhance the functional properties and antioxidant activity of lutein [57].

### 5.4. Targeted Controlled Release of Zein-Loaded Lutein Nanoparticles

Lutein is released in large quantities into the gastrointestinal tract through chewing and enzymatic action in the mouth. It disperses in the digestive tract with dietary fats, pancreatic fluid, and bile; dissolves in the mixed micellar phase formed in the small intestine; and is then absorbed by epithelial cells and packaged into lipoproteins for delivery into the bloodstream. However, due to its low solubility, lutein struggles to reach the digestive tract and be absorbed by the small intestine’s epithelial cells, resulting in low absorption efficiency and bioavailability. Encapsulation methods are used to address this. The ideal delivery vector must be stable in the product’s matrix, protect the active molecules from degradation or loss during storage, and ensure significant release of the active molecules at the appropriate site [74]. Food-grade protein carriers offer a natural encapsulation material with nutritional value. However, many protein-based encapsulants are prone to rapid digestion or degradation before reaching the desired site in the gastrointestinal tract unless the protein is modified or coated with a secondary indigestible material [75]. Zein-based colloidal carriers present unique advantages, such as low cost and potentially reduced accessibility to digestive enzymes. As a by-product of corn biodiesel and starch production, Zein is abundant and recognized as a safe protein source [76]. Using in vitro simulated gastrointestinal digestion models can conveniently, quickly, and efficiently simulate human digestion and absorption of food, allowing for better control of lutein digestion and absorption by mimicking gastrointestinal fluid, thus achieving precise targeted drug delivery. Cheng et al. found that encapsulating lutein in Zein improves its digestive stability and reduces micellization efficiency. Under simulated gastric conditions, physiological ion concentrations caused zein nanoparticles (ZNPs) to aggregate into relatively inaccessible clusters to enzymes, resulting in a significant portion of ZNPs remaining undigested and protected in the stomach. Under simulated intestinal conditions, all ZNPs, including these clusters, were completely digested into shorter peptides, enabling lutein to achieve targeted controlled release and complete digestion in the intestine, significantly improving lutein absorption rates [44]. Stability and release studies in SGF and SIF by Jiao et al. showed that pepsin-hydrolysed corn alkyd protein nanocarriers improved digestive stability and facilitated lutein release under gastrointestinal digestion conditions. The results indicated that the cumulative release of lutein in LZHN was higher than in Zein in both SGF and SIF. Additionally, the release of lutein in alkaline intestinal fluid was superior to that in acidic gastric fluid. These results may be due to Zein hydrolysate exhibiting greater hydrophilicity than Zein, leading to better solubility and dispersion in SGF and SIF, allowing lutein loaded in the Zein hydrolysate to be easily released from the nanoparticles. Consequently, the cumulative release of lutein is high, especially in intestinal fluid, demonstrating the targeted controlled release of lutein [54].

### 5.5. Bioaccessibility of Zein-Loaded Lutein Nanoparticles

Lutein, a naturally occurring carotenoid, is also known as plant photoreceptor pigment and spot pigment, which can effectively promote and maintain human health [77,78]. Lutein effectively filters blue light to prevent retinal damage and reduces the incidence of age-related macular degeneration (AMD), cataracts, and heart disease. However, as a long-chain hydrophobic molecule with multiple conjugated double bonds, lutein has very poor water solubility and stability, resulting in low bioavailability [79,80]. Researchers have extensively studied delivery systems, such as liposomes, nanoparticles, and emulsions, to address this deficiency in transporting lutein in the food and pharmaceutical fields. Studies have shown that when lutein is administered as nanoemulsions, its bioavailability (112.6 ng/mL) is significantly higher than that of unencapsulated lutein (48.6 ng/mL) and mixed micelles (68.5 ng/mL). The tissue distribution pattern of lutein nanoemulsions indicates higher lutein accumulation in the liver (2.80 and 1.70 fold) and eyes (1.91 and 1.48 fold) compared to free lutein and mixed micelle-fed groups [81].

The bioaccessibility of lutein mainly refers to the ability of lutein to be released and absorbed once it enters the body. As a fat-soluble vitamin, lutein must dissolve and be absorbed with lipids, severely limiting its bioaccessibility [82]. During absorption, lutein forms micelles with lipids, which are then absorbed in the intestines and transported through the lymphatic and blood systems to the liver, where it is distributed throughout the body. Studies have shown that a ternary composite system of lutein provides excellent stability in the simulated gastrointestinal tract, allowing for effective release into the intestines. Additionally, the Ussing-chamber system demonstrated that ternary composite nanoparticles of lutein more easily penetrate the intestinal wall and enter the bloodstream, thereby improving the absorption and utilization of lutein [74].

Bioaccessibility determines the total amount of a compound that can be absorbed during digestion, while bioavailability refers to the portion that enters systemic circulation to participate in physiological functions [83]. The addition of modifiers can increase the bioaccessibility of lutein by reducing cytotoxicity and improving nanoparticle absorption efficiency through the cell lipid bilayer, enhancing internalization. Some researchers have used NCM460 cells (human normal colon epithelial cells) to evaluate the cytotoxicity of unencapsulated lutein and lutein-loaded zein/SPSS nanoparticles at different concentrations [55]. Even at 100 μg/mL, cell viability remained greater than 85% after 24 h of incubation, indicating that these nanocarriers are non-toxic and biocompatible, significantly improving lutein’s bioaccessibility and maximizing its absorption. This finding aligns with previous findings where curcumin’s bioaccessibility in SIF increased to approximately 25% when encapsulated with ternary nanoparticles of zein–alginate–fish gelatin [84].

Other researchers evaluated the effects of free lutein, zein nanoparticles, lutein-loaded Zein nanoparticles, lutein-loaded zein/sophoroladiin nanoparticles, and zein/sophoroladiin nanoparticles on the viability of NCM460 cells [60]. The results showed that lutein in zein/sophorolipid nanoparticles was essentially non-toxic. Furthermore, using sophorolipid or Zein increased cell viability, demonstrating good biocompatibility. Additionally, zein/chondroitin sulfate nanoparticles were non-toxic to cells, confirming the non-toxicity and bioaccessibility of these materials. Ji et al. reported that insulin-containing zein/carboxymethylated short-chain linear starch nanoparticles were non-toxic to Caco-2 cells, indicating good bioaccessibility [85]. Shwetha et al. evaluated the bioavailability and intestinal absorption capacity of zein–alginate–phosphatidylcholine nanoparticles encapsulating lycopene and lutein using in vitro digestion and human intestinal Caco-2 cell models. The results showed that the bioavailability, cellular uptake, and basolateral secretion of lycopene and lutein in the nanoparticles were significantly enhanced compared to micelle carotenoids (the direct dietary absorption model) [86].

**Figure 5:**
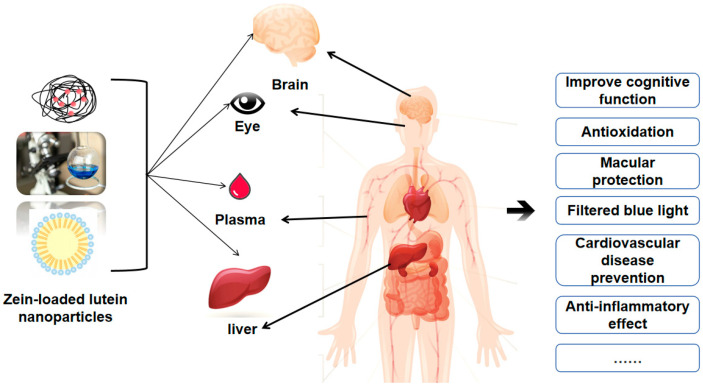
Zein-loaded lutein nanoparticles are targeted for delivery to the brain, eyes, liver, and plasma to exert the biological activity of lutein.

Currently, the evaluation of zein-loaded lutein nanosystems primarily focuses on changes in their physical and chemical properties, such as morphological characteristics, characterization, stability, encapsulation efficiency, and simulation of human digestion, as shown in Table 3. The research is not very in-depth, and relatively few studies involve cellular and animal models. However, as a non-toxic, green plant protein, Zein has the potential to construct a targeted lutein delivery system comparable to those made from materials like chitosan and lipids. Figure 5 shows that zein-loaded lutein nanoparticles can target the brain, eyes, liver, and other parts of the body to enhance the biological functions of lutein. The smaller particle size, biocompatibility, and stabilizing system of these nanoparticles are likely to prolong the contact and duration of lutein with organs, thereby promoting its uptake and utilization in the body [87,88,89,90].

## 6. Conclusions

The selection of nanosystems constructed by Zein with self-assembly properties for the protection and delivery of lutein is a recent technological advancement. Methods for loading lutein onto Zein include nanolipidation, nanoparticle preparation by anti-solvent method, and supercritical fluid enhanced solution dispersion (SEDS), which, combined with nanosizing techniques such as high-pressure microjet flow, have yielded promising results. The structure and properties of Zein nanoparticles can be further enhanced by modifying Zein through techniques such as acid-induced deamidation, enzymatic modification, and glycosylation. Compared to Zein alone, Zein complexes can enhance the bioactivity, stability, and solubility of lutein, improving its targeted release capability and bioaccessibility. Characterization and analysis techniques for zein-loaded lutein nanoparticles include particle size, PDI, zeta potential, and microscopic morphology measurements. The overall composition and structural properties are analyzed using FT-IR, XRD, and NMR techniques. In investigating functional properties, the target-controlled release mechanism of zein-loaded lutein and its in vivo metabolism have become research hotspots in recent years. In summary, the research and application of Zein and its derivatives for supporting lutein nanosystems have garnered increasing attention in food and medicine. This nanodelivery system is significant for the development of novel lutein-based nutritional and functional foods. Future trends should focus on the research and application of large-scale industrial production and the economic feasibility of zein-based lutein nanodelivery systems.

## Figures and Tables

**Table 1:** Zein content distribution table.

Type	Concentration	Molecular Mass/kDa
α-zein	75–85%	19, 22
β-zein	10–15%	24, 22, 14
γ-zein	5–10%	16, 27, 50
δ-zein	1–5%	10, 18

**Table 2:** Characterization method of lutein loaded with Zein.

Structural Characterization	Method	Application	Reference
Encapsulation efficiency	Chromatography–mass spectroscopy	Hu et al. measured the encapsulation rate of Zein-LUT by liquid chromatography at 83.15%. De Boer et al. measured the encapsulation rate of nanoparticles by ultraviolet spectrophotometer.	[49,58]
Nanoparticle size	Particle size and zeta potential analysis	The particle size of zein-loaded lutein was measured by a particle size analyzer, and the surface zeta potential was 27.4–29.4 mv. The particle size potential analysis could reflect the dispersion and stability of the nanoparticles.	[58]
Microstructure	TEM	Chuacharoen observed that the surfactant-containing particles were spherical with a rough surface, and some of the particles were connected in the surfactant lattice. The size of the nanoparticles without surfactants was smaller and more spherical but uneven in size and more prone to agglomeration.	[59]
	SEM	Jiao et al. observed that Zein and its derived peptides were spherical and that the size of Zein was larger than that of its derived peptides. When lutein is loaded into Zein nanoparticles, the size of the nanoparticles decreases and aggregates.	[53]
	AFM	Cheng et al. observed a variety of distinct two-dimensional amorphous components, particles ranging in size from 10 to 700 nm in diameter, and particle aggregates.	[45]
Structural characteristic	FTIR	Liu et al. analyzed the structural information of the samples and found that more hydrogen bonds were formed after the hydrolysis of Zein, and the secondary structures of hydrolysates obtained by different enzymes were significantly different.	[42]
	XRD	Li et al. found that after encapsulation, the typical diffraction peak of lutein did not appear in the ZS-LNPs spectrum of nanoparticles formed by Zein, indicating that lutein may be distributed in the nanoparticles in an amorphous manner.	[55]
Structural stability	PDI	Li et al. found that the size of Zein nanoparticles Z-LNP and ZS-LNP was significantly different (*p* < 0.05), indicating the interaction between Zein and lutein, while soy protein existed in the form of single molecules and associated aggregates.	[55]
	Protein modification	Accessories: ascorbic acid, tea saponin, soy protein, sophorolipid, glucosamine, lecithin, Pluronic F127 emulsifier, etc.	[55,56,57,58,59,60]

**Table 3:** Functional properties of zein-supported lutein.

Nature	Modified Product	Application	Reference
Stability	Ascorbic acid	The degradation rate of lutein by photodegradation was significantly decreased	[58]
	The Planik F127	After 10 h of ultraviolet radiation, more lutein was still retained	[59]
	Tea saponin	It has good stability under high-salt concentration	[56]
Solubility	Glucose	Zein binds to glucosamine through an enzymatic reaction	[57]
	Soybean polysaccharide	The solubility of lutein was increased by more than 30 times	[55]
Oxidation resistance	Glucosamine chitosan	The encapsulation of Zein and glycosylated Zein enhanced the antioxidant activity of lutein in vitro	[57]
		The glycosylated Zein carrier protects and enhances the antioxidant activity of lutein	[87]
Targeted controlled release	Biological enzyme hydrolysis	Protect lutein from massive degradation or loss in the simulated artificial gastric fluid environment and complete digestion in the simulated artificial intestinal fluid, allowing lutein to complete targeted, controlled release and digestion in the intestine	[55]
Biological accessibility	Alginate	Compared to direct dietary uptake of lutein, the Zein carrier significantly increased lutein bioavailability, cellular uptake, and basolateral secretion	[55]

## Data Availability

No new data were created or analyzed in this study. Data sharing is not applicable to this article.
